# Suspected pleuroparenchymal fibroelastosis relapse after lung transplantation: a case report and literature review

**DOI:** 10.1259/bjrcr.20190040

**Published:** 2019-11-15

**Authors:** Edoardo Rasciti, Alessandra Cancellieri, Micaela Romagnoli, Andrea Dell'Amore, Maurizio Zompatori

**Affiliations:** 1 Department of Radiology, S. Orsola Malpighi University Hospital, Bologna, Italy; 2 Department of Pathology, S. Orsola Malpighi University Hospital and Maggiore Hospital, Bologna, Italy; 3 Interventional Pneumology Unit, S. Orsola Malpighi University Hospital, Bologna, Italy; 4 Pulmonology Unit, Cà Foncello Hospital, AULSS2 Marca Trevigiana, Treviso, Italy; 5 Department of Cardio-Thoracic Surgery, S. Orsola Malpighi University Hospital, Bologna, Italy; 6 MULTIMEDICA IRCCS S. Giuseppe Hospital, Milan, Italy

## Abstract

Pleuroparenchymal fibroelastosis (PPFE) is a very rare lung disease characterized by dense fibrous thickening of the visceral pleura and intraalveolar fibrosis containing prominent elastosis, with typical upper lobe predominance. PPFE usually shows progressive restrictive ventilatory impairment refractory to medical treatment; bilateral lung transplantation currently remains the only available therapeutic option. In this report, we describe a case of suspected PPFE relapse after lung transplantation that, to our knowledge, has never been described in the medical literature.

A 48-year-old male with idiopathic pleuroparenchymal fibroelastosis underwent a bilateral lung transplant in our department. 8 months later, he presented with progressively worsening clinical condition, his respiratory state gradually deteriorated. High-resolution CT again showed bilateral diffused parenchymal consolidations, with prevalence in the upper lobes and subpleural regions. A PPFE relapse was therefore suspected, so he was listed for lung retransplantation, which was performed ten months after the first transplant. Histopathological analysis of the second explanted lung again confirmed the diagnosis of PPFE. The case highlights the possibility of PPFE relapse after lung transplantation, that may add to the increasing evidence of an underlying auto-immune mechanism contributing to its pathogenesis.

Pleuroparenchymal Fibroelastosis (PPFE) is a rare interstitial lung disease that is characterized by upper lobes pleural thickening and subpleural fibrosis.^1^ Differently from other more common patterns of pulmonary fibrosis, like usual interstitial pneumonia (UIP) and non-specific interstitial pneumonia (NSIP), PPFE typically shows prominent elastotic fibrosis of the pleura and subjacent lung, with sparing of the parenchyma distant from the pleura.^2^

PPFE pathogenesis is still poorly understood, as it can either be idiopathic or secondary to many underlying diseases. In the updated American Thoracic Society–European Respiratory Society classification of the idiopathic interstitial pneumonias (IIPs), idiopathic PPFE (iPPFE) has been recognized as a separate entity, and categorized as a rare form of interstitial pneumonia.^1^

The clinical course of this severe disease is characterized by slowly progressive restrictive ventilatory impairment. No effective medical treatment has been identified, as both secondary and idiopathic form of PPFE are refractory to steroids or immunosuppressive drugs. As such, bilateral lung transplantation is the only currently available therapeutic option.^2^

In this report, we describe a case of suspected iPPFE relapse after lung transplantation.

## Case presentation

**Figure 1.**
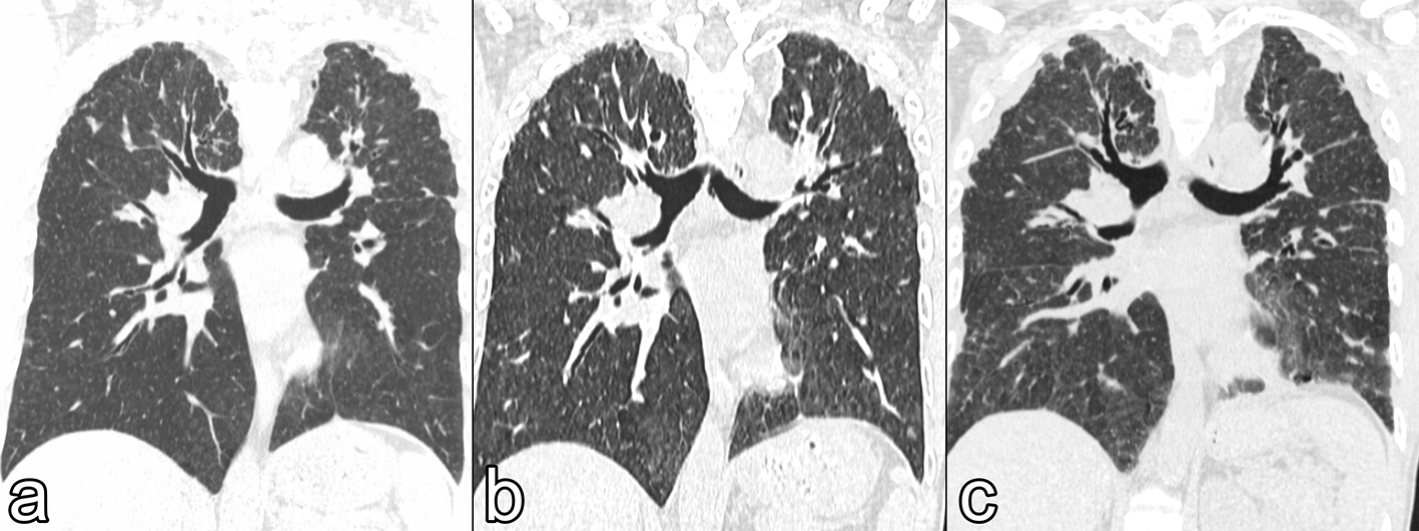
Coronal HRCT images showing the progression of PPFE on the native lung. (a) Shows mild bilateral apical pleural thickening with some subpleural reticulation. (B) After 1 year, HRCT shows a moderate increase of the irregular pleural thickening, together with traction bronchiectasis and early upper-lobe volume loss; at this stage, surgical lung biopsy confirmed the diagnosis of PPFE. (c) 2 years later, HRCT shows a further increase in irregular apical pleural thickening with subpleural reticulation and traction bronchiectasis; upper-lobe volume loss is more marked on the left lung. HRCT, high-resolution CT; PPFE, pleuroparenchymal fibroelastosis.

A 48-years-old male with iPPFE was referred to our department for a lung transplantation consultation. He received a diagnosis of iPPFE 2 years before with surgical lung biopsy after a few months of cough and dyspnea (Figure 1). His past medical history was silent, except for a previous Ravitch procedure for pectus excavatum.

At admission, the patient presented with wheezes, diffuse inspiratory crackles and reduced vescicular murmur, predominantly in the upper lobes. SpO_2_ was 94% at rest.

Chest high-resolution CT (HRCT) showed bilateral parahilar fibrotic interstitial thickening with architectural distortion, dorsal pleural thickening and subpleural consolidations, predominantly in the upper lobes (Figure 1). Pulmonary function testing (PFTs) progressively worsened; 3 months later, PFTs showed severe restrictive ventilatory impairment [forced vital capacity (FVC), 0.93 L; % FVC, 23%] with poor diffusing capacity of carbon monoxide (%DLCO, 21%), so he was listed for lung transplantation. The patient underwent a bilateral lung transplant 3 months after being included on the waiting list.

**Figure 2.**
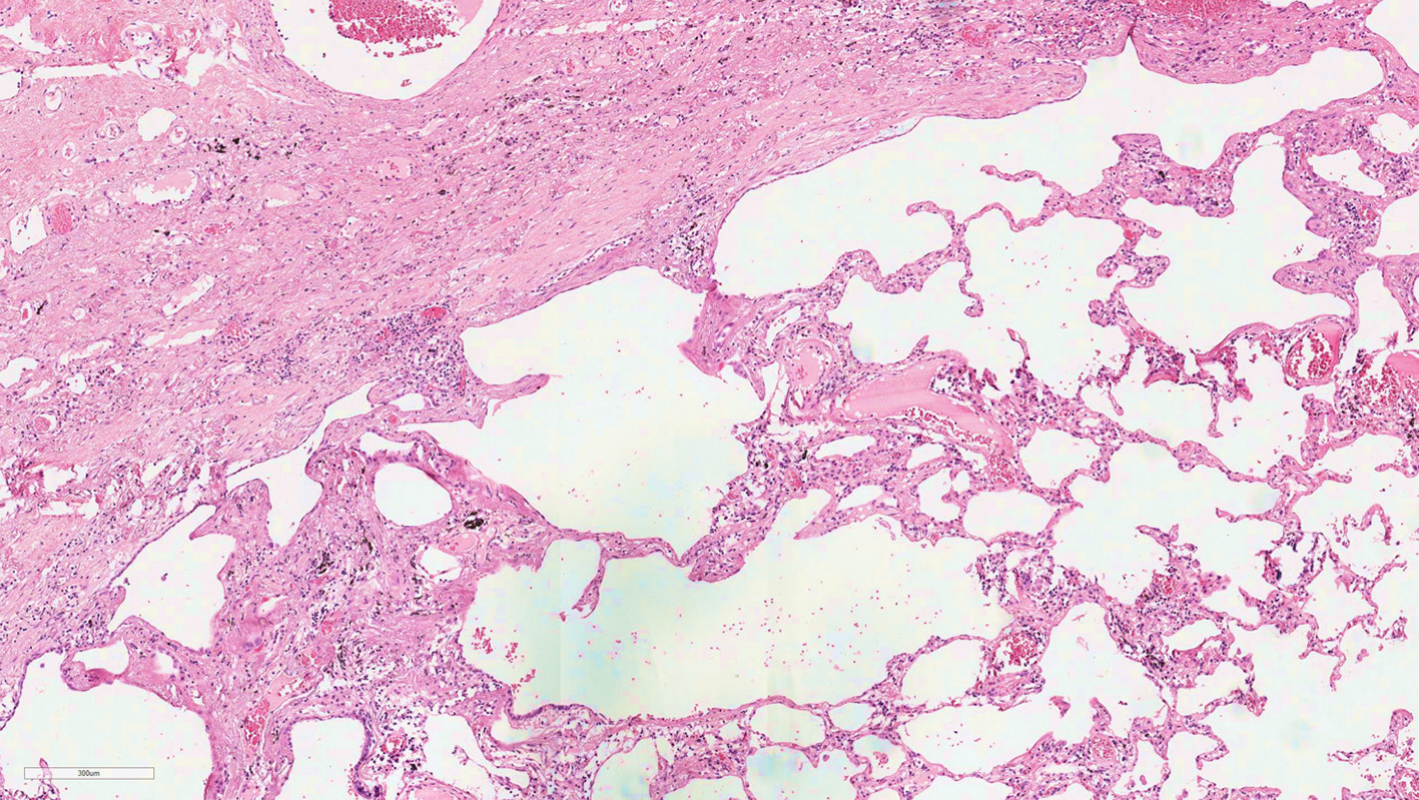
Histopathological analysis of the native lung shows diffuse areas of dense and homogeneous fibrosis, rich in elastic fibres, and partially extending into alveolar and interlobular septa. Fibroblastic foci at the edge of the fibrosis were incospicuous or absent (H&E).

The postoperative course was uneventful, and the patient was discharged on postoperative day 20. The histopathological analysis confirmed the diagnosis of iPPFE, as it showed diffuse areas of dense and homogeneous fibrosis, rich in elastic fibers, partially extending into alveolar and interlobular septa; fibroblastic foci at the edge of the fibrosis were incospicuous or absent (Figure 2).

**Figure 3.**
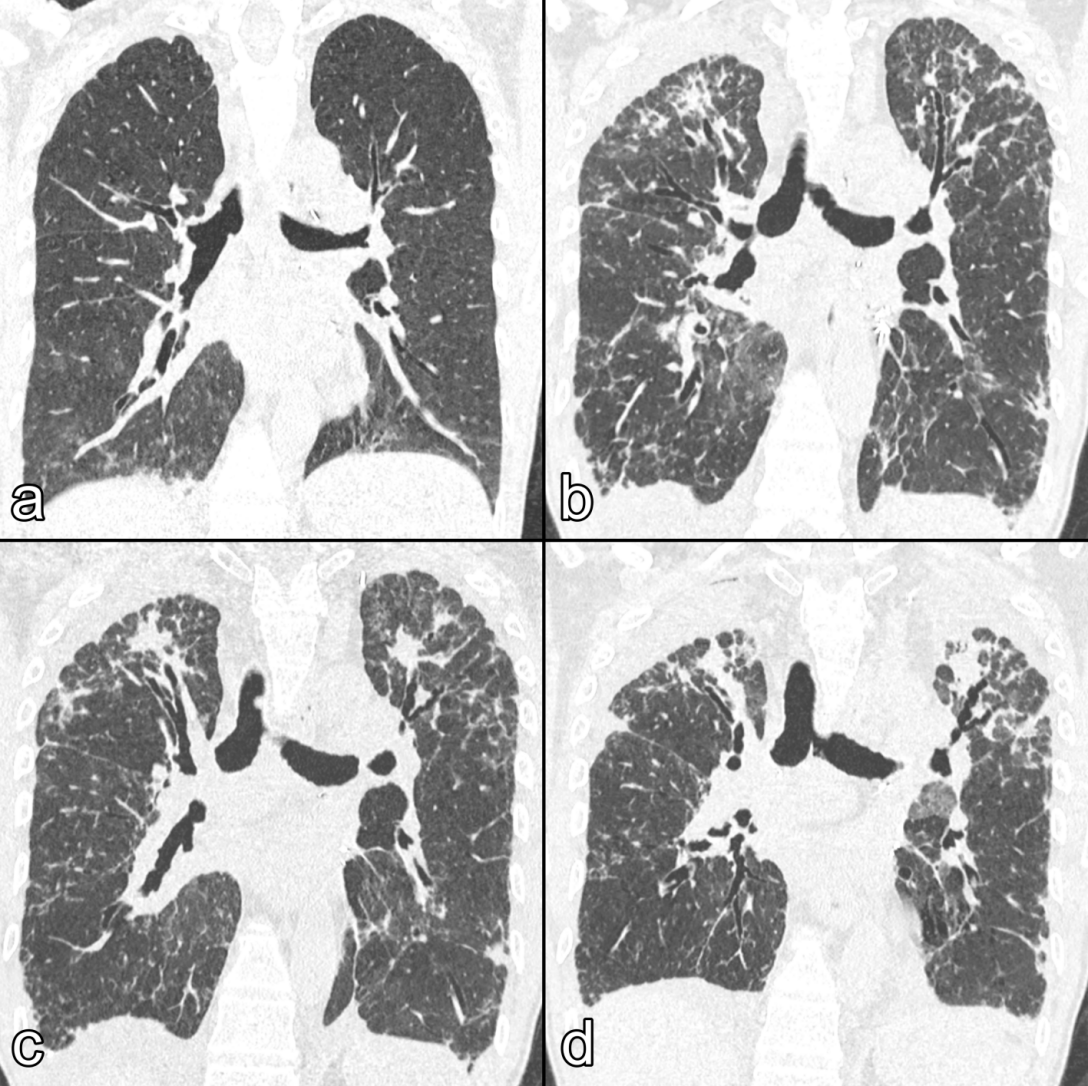
Coronal HRCT images showing the progression of the PPFE relapse after the first bilateral lung transplantation. (a) The first post-operative HRCT shows regular outcome of the transplantation and no signs of bronchial obliterative syndrome. (b) After 4 months, HRCT shows bilateral diffused parenchymal consolidations, with prevalence in the upper lobes and in the subpleural regions, together with some reticular opacities, traction bronchiectasis and mild pleural thickening. (C) 1 month later, HRCT shows increase in both the parenchymal consolidations and pleural thickening, together with mild upper-lobe volume loss. (D) After 3 months, HRCT shows marked pleural thickening together with traction bronchiectasis and severe lung volume loss; at this stage, the patient underwent the second lung transplantation. HRCT, high-resolution CT; PPFE, pleuroparenchymal fibroelastosis.

8 months later, however, he presented with progressively worsening clinical condition. His respiratory state gradually deteriorated (FVC, 1.39 L; % FVC, 33%; FEV1, 1.18 L; % FEV1, 34%), the laboratory tests showed no abnormalities and the screening for anti-human leukocyte antigen antibodies was negative. HRCT again showed bilateral diffused parenchymal consolidations, with prevalence of the upper lobes and in the subpleural regions, together with some reticular opacities, traction bronchiectasis and mild pleural thickening, with a radiologic pattern similar to the original PPFE; no signs of bronchial obliterative syndrome (BOS) were detected (Figure 3). A PPFE relapse was therefore suspected.

Oxygen was started and his immunosuppressive therapy was increased, with no changes.

After 1 month, his lung function steadily declined, so he was listed for lung retransplantation, which was performed 10 months after the first transplant (Figure 3). He did not show any signiﬁcant postoperative complications and he was discharged on postoperative day 23.

**Figure 4.**
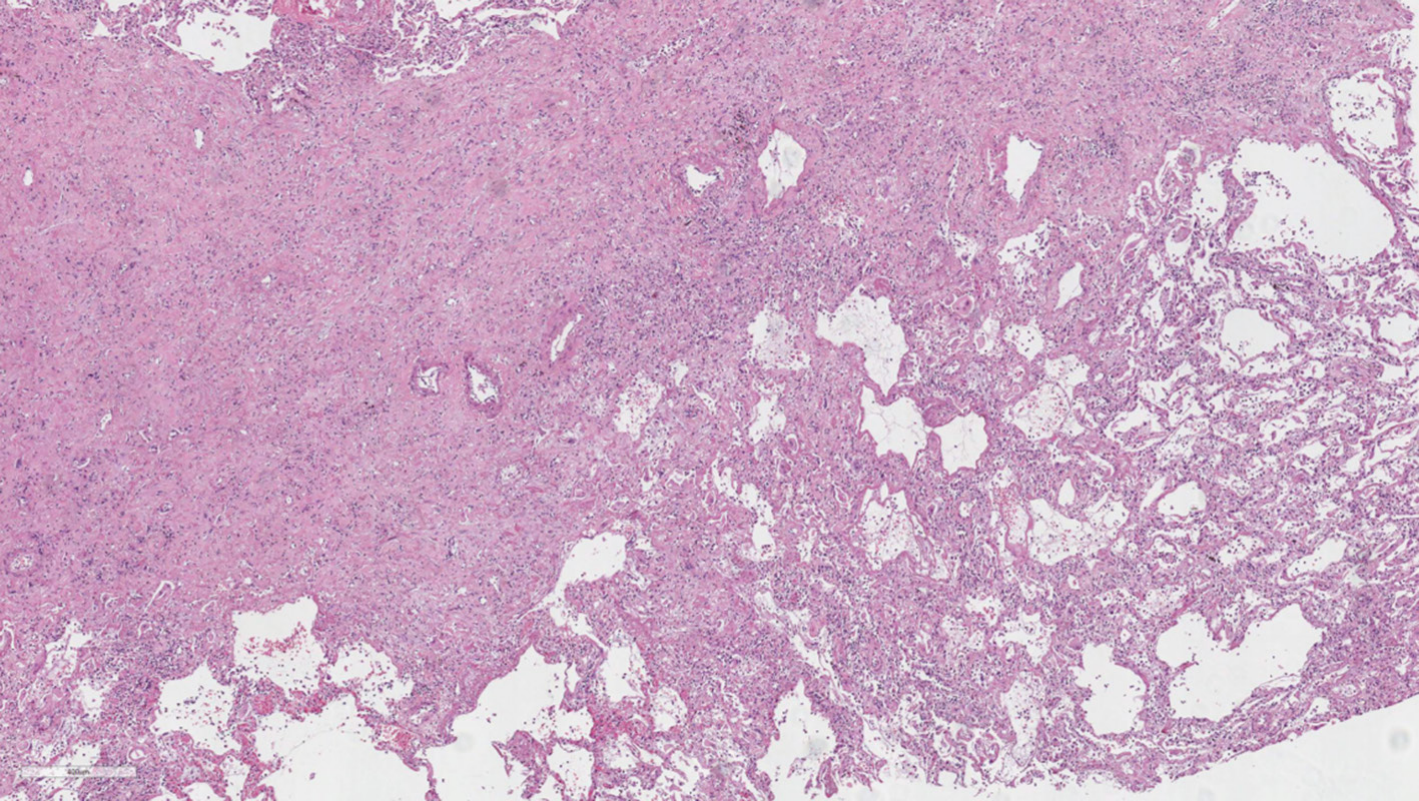
Histopathological analysis of the second explanted lung shows fibrosis with a more diffuse pattern of distribution, predominantly in the subpleural regions, with a prominent elastotic component. The residual parenchyma showed features of acute lung injury and foci of obliterative (constrictive) bronchiolitis (not shown) (H&E).

Histopathological analysis of the second explanted lung again confirmed the diagnosis of PPFE, as it revealed fibrosis with a more diffuse pattern of distribution, predominantly in the subpleural regions, with a prominent elastotic component, together with foci of obliterative bronchiolitis (Figure 4). 12 months later, he is doing well without oxygen supplementation.

## Discussion

PPFE is a very rare clinicopathological entity consisting of a form of progressive pulmonary fibrosis involving the pleura and subpleural lung parenchyma, with upper lobe predominance.^1^

Amitani et al first described some form of idiopathic pulmonary upper lobe fibrosis in 1992,^3^ while Frankel et al were the first to propose such entity as PPFE in 2004 ^4^. According to Watanabe et al, only around 100 cases have been reported in the literature.^2^

Chest HRCT shows dense subpleural consolidation with pleural thickening, traction bronchiectasis, architectural distortion, and volume loss predominantly in the upper lobes.^5,6^

A final diagnosis of PPFE may require surgical lung biopsy, showing peculiar histopathology consisting of marked visceral pleural thickening and prominent subpleural fibrosis with both elastic tissue and dense collagen.^7^

PPFE may have a long, asymptomatic subclinical stage, in which lesions are limited to the apex,^2^ whereas in most cases it manifests with dyspnea, recurrent respiratory infections, pneumothorax, and disease progression with death in 40% of cases.

The clinical course of this disease is slowly progressive (median survival 11 years), refractory to steroids or immunosuppressive drugs, with lung transplantation being the only currently available therapeutic option.^2^

PPFE is a complex disease that can either be idiopathic or secondary to many underlying diseases or conditions, *e.g.* anticancer chemotherapy, bone marrow or stem cell-transplantation, occupational dust exposure (asbestos, aluminum).^4^

No connection with gender or smoking history has been detected so far. Patients with PPFE frequently show a flattened thoracic cage,^2^ as in our case.

As PPFE pathogenesis is still poorly understood, a contributing auto-immune mechanism has been proposed by some authors: PPFE has been reported in siblings, in a pair of parents and child and in patients with other autoimmune diseases like ankylosing spondylitis, ulcerative colitis, and psoriasis.^8^

Recently, PPFE has been also identified as a phenotype of chronic rejection of lung transplantation, as it represents a major histopathological correlate in restrictive allograft syndrome (RAS).^8–10^ Transplantation-associated PPFE seems to have some peculiar histopathological differences from iPPFE, and appears to have a poorer prognosis than other secondary causes of PPFE.^8,11,12^

Konen et al^10^ reported upper lobe fibrosis in a range of 18–72 months (mean 42) after lung transplantation, suggesting that it could be a late-onset complication.

Accordingly, Mariani et al^13^ reported six cases of PPFE developing 2–13 years (average 5.3) after lung transplantation or hematopoietic stem cell transplantation.

In our case, only 5 months passed between the transplantation and the first clinical symptoms of lung function decline, and only 10 between the two lung transplantations. This may suggest the contribution of an underlying process accelerating the development of PPFE in the transplanted lung.

In conclusion, our case highlights the possibility of PPFE relapse after lung transplantation, that may add to the increasing evidence of an underlying auto-immune mechanism contributing to its pathogenesis. More studies are needed to further understand PPFE pathogenesis and to identify potential alternative effective treatments; more importantly, potential causes of lung transplantation failure for disease relapse need to be addressed, to better identify potential candidates to transplantation.

## Learning points

1. Pleuroparenchymal Fibroelastosis is a rare and progressive form of pulmonary fibrosis with upper lobe predominance, and its pathogenesis is still poorly understood
2. The case highlights the possibility of PPFE relapse after lung transplantation, adding to the increasing evidence of an underlying auto-immune mechanism contributing to its pathogenesis.
3. PPFE may also represent a histopathological and radiological pattern of chronic lung allograft dysfunction after lung transplantation.
